# Influence of Strength, Power, and Muscular Stiffness on Stroke Velocity in Junior Tennis Players

**DOI:** 10.3389/fphys.2020.00196

**Published:** 2020-03-06

**Authors:** Joshua Colomar, Ernest Baiget, Francisco Corbi

**Affiliations:** ^1^National Institute of Physical Education (INEFC), University of Barcelona, Barcelona, Spain; ^2^Sports Science Department, Academia Sánchez-Casal, Barcelona, Spain; ^3^Sports Performance Analysis Research Group (SPARG), University of Vic - Central University of Catalonia, Vic, Spain; ^4^National Institute of Sport and Physical Education (INEFC), University of Lleida, Lleida, Spain

**Keywords:** serve, forehand, backhand, speed, testing

## Abstract

**Objective:**

The main aim of this study was to establish the relationship between strength, power characteristics, individual muscle stiffness, international tennis number (ITN), and stroke velocity (StV) in junior tennis players.

**Methods:**

Twenty one junior male tennis players (mean ± SD; age, 17.0 ± 0.8 years; height, 1.8 ± 0.1 m; body mass, 72.3 ± 5.8 kg; BMI 22.1 ± 1.5 kg/m^2^), with an ITN ranging from 2 to 4, performed measurements regarding muscle stiffness of selected muscles involved in tennis strokes. StV (serve, forehand, and backhand), strength (maximum isometric strength) and power (medicine ball throws, squat jump, countermovement jump, and bench press) measurements were also performed (ICC = 0.803–0.998; CV = 0.3–6.4).

**Results:**

Moderate inverse correlations were found between serve velocity (SV) and ITN (*r* = −0.43; *p* = 0.05), and large positive correlations were observed between pectoralis majoris stiffness (PMStiff) (*r* = 0.53; *p* = 0.01), isometric wrist flexion (*r* = 0.58; *p* = 0.006) and ITN, respectively. PMStiff was moderately inversely correlated to forehand velocity (FV) (*r* = −0.45; *p* = 0.03) and gastrocnemius (GStiff) and infraspinatus stiffness (IStiff) positively to SV (*r* = 0.45; *p* = 0.04; *r* = 0.42; *p* = 0.05). No significant correlations were found regarding strength and power measurements.

**Conclusion:**

Greater stiffness values may enhance StV, especially when transferring power from lower to upper body. On the other hand, high scores could interfere in technical parameters that are key for velocity production in complex tennis strokes. Strength and power values proved to correlate poorly to StV in this particular sample of junior tennis players, possibly due to the multifactorial nature of tennis strokes and the possibility that they become more important as age and level increase.

## Introduction

Today’s tennis is considered as a fast-paced, explosive and highly dynamic sport (Roetert et al., 2009b; Fernández-Fernández et al., 2014). High hitting velocities, specifically in the serve, can decide the game and are directly related to the tennis player’s level (Gillet et al., 2009; Ulbricht et al., 2016). This action has been considered the most important shot, due to the possibilities to dominate the rally or win the point directly through an ace (Gillet et al., 2009; Kovacs and Ellenbecker, 2011). On the other hand, groundstroke velocity has received less attention by literature, although some data on the matter suggest an increased hitting velocity when comparing professional and youth high-performance players (Landlinger et al., 2012). Achieving higher velocity production in strokes could be an important factor on which players may benefit in order to improve performance and achieve higher competitive levels. Moreover, tennis strokes are considered highly complex motor skills which require force production and the ability to transfer these forces throughout the entire body in what is known as the kinetic chain (Kibler, 2009, 2014). Thus, further knowledge around specific determinants of stroke velocities and how they influence performance could be of interest for practitioners.

Because of the aforementioned characteristics, it is commonly been accepted that these strokes are affected by several parameters such as technique (Roetert et al., 2009a, b), anthropometrics (Söğüt, 2014; Bonato et al., 2015), strength, power (Baiget et al., 2016; Fett et al., 2018; Palmer et al., 2018) biological (Sanchís-Moysi et al., 2010), and range of movement (ROM) characteristics (Palmer et al., 2018), making the action of a multi-factorial nature. Several studies have focused on biomechanical and kinematic factors influencing hitting performance (Elliott et al., 1995; Roetert et al., 2009a, b), establishing the speed of the racquet head, internal rotation of the upper arm, wrist flexion and moment of ball impact as some of the major contributors to generate velocity (Elliott et al., 1995). Also, anthropometrics such as height and body mass have been related positively with serve speed in professional and young players (Söğüt, 2014; Bonato et al., 2015; Fett et al., 2018). Regarding strength values, literature traditionally has focused on analyzing isokinetic data at certain joint positions and degrees that mimic the serve action (Ellenbecker and Roetert, 2003), obtaining moderate positive correlations especially on those positions that resemble the serve motion. More recently, investigations have also aimed to assess strength values adding maximal isometric strength testing to experimental methods, especially in the shoulder complex (Cools et al., 2014; Baiget et al., 2016; Fett et al., 2018; Hayes et al., 2018). When assessing SV, it has been accepted as a contributor to velocity production, but few investigations have aimed to the relationship between maximal isometric strength and forehands or backhands. Further research regarding groundstrokes could be of interest as previous studies have shown a strong relationship between isometric strength and performance (Baiget et al., 2016). When focusing on dynamic strength, some interesting data has recently been analyzed, indicating upper body strength and power as important contributors of the junior tennis player’s serve (Fett et al., 2018; Palmer et al., 2018). Nevertheless, as the majority of investigations have established strength and power as contributors to SV in elite players, it seems interesting to further focus on these measurements regarding young competitive players and especially analyzing the groundstrokes. Taking into account these actions as determinant factors differentiating elite from sub-elite players (Landlinger et al., 2012), it seems important to study associations between strength and power characteristics and groundstrokes, as we find regarding SV in literature.

Furthermore, the influence of complex neuromuscular factors has hardly been studied in relation to any tennis specific stroke. Enhanced values of mechanical stiffness, that can be defined as the resistance of an object or body to deformation or change in length (Brughelli and Cronin, 2008), have been suggested as beneficial for actions that rely on the stretch shorten cycle (SSC) such as jumping, sprinting or agility (Brughelli and Cronin, 2008). Due to the greater capacity of a compliant structure to absorb and re-use rapidly greater amounts of elastic energy for a given force (Kalkhoven and Watsford, 2018), this quality could be beneficial or have influence on tennis strokes, as they are complex skills that involve SSC actions in the entire kinetic chain. On the other hand, an increased or non-sufficient level of the mentioned stiffness could interpose technical aspects or the capacity to produce velocity to the stroke (Brazier et al., 2017). Because of this, studies have started to investigate neuromechanical factors such as individual muscle stiffness and their contribution to performance aspects (Sheehan et al., 2018). The majority of investigations on stiffness have aimed at actions involving the lower body (Pruyn et al., 2014; Kalkhoven and Watsford, 2018), making this phenomenon still unclear when speaking of how it affects predominantly upper body motions. Added to this, investigations have aimed to establish specific predictors of tennis actions, yet to the best of our knowledge, none concerning the relationship between muscle stiffness characteristics and stroke velocity (StV), especially on groundstrokes and junior tennis players.

In short, the influence of specific strength and power parameters on StV, especially groundstrokes, and how muscle mechanical properties affect dynamic actions seems of importance for professionals. Therefore, the aim of this study was to examine the relationship between strength and power characteristics, individual muscle stiffness values, international tennis number (ITN) and StV in competitive junior tennis players. Our working hypothesis was that a strong positive association will exist between strength and power characteristics, ITN and all strokes, as seen previously in SV (Baiget et al., 2016; Fett et al., 2018; Hayes et al., 2018; Palmer et al., 2018). Also, due to the beneficial effects of enhanced stiffness in explosive actions (Brughelli and Cronin, 2008), a higher level of this property in the muscle groups tested will correlate to faster StV.

## Materials and Methods

### Participants

Twenty-one junior male tennis players (mean ± SD; age, 17.0 ± 0.8 years; height, 1.8 ± 0.1 m; body mass, 72.3 ± 5.8 kg; BMI 22.1 ± 1.5 kg/m^2^) with an ITN ranging from 2 to 4 (advanced level) participated in this study. *A priori* power analysis for a Pearson correlation was conducted in G^∗^power to estimate a sufficient sample size. With the alpha level set at 0.05, using a large target effect size (ES) of 0.6, a power of 0.80 and two tails, it was determined that 19 subjects would be needed. The player’s ITN was established by the consensus of three coaches accredited with RPT (Registro Profesional de Tenis) level 3, following the ITN Description of Standards (ITN, 2019). Subjects had a weekly volume of training of 25 h/week^–1^ of which 5 accounted for fitness training and 20 for technical and tactical sessions. The mean training background of the players was 10.1 ± 1.7 years, which focused on tennis-specific training (i.e., technical and tactical skills), aerobic and anaerobic training (i.e., on- and off-court exercises), and strength training. Inclusion criteria for all subjects required each participant to have a minimum of 1 year experience in strength training and 5 years of tennis training and competition. Participants were excluded from the study if they had history of upper extremity surgery, shoulder, back or knee pain and/or rehabilitation for the past 12 months. All subjects were informed in advance about the characteristics of the study and, before their participation, the participants or their legal tutors, in the case of being underage, voluntarily signed an informed consent. The study was conducted following the ethical principles for biomedical research with human beings, established in the Declaration of Helsinki of the AMM (2013) and approved by the Ethics Committee of the Catalan Sports Council (26/2018/CEICEGC).

### Experimental Design

This was a cross-sectional laboratory study with uninjured participants. The collection of data took place in May during a normal in-season training week in groups of 4 or 5 players and on 4 separate testing sessions, executed from 8 a.m. to 2 p.m. approximately, before the player’s afternoon normal technical-tactical training. On session 1, participants were assessed for 1 repetition maximum (1RM) on the bench press exercise. On session 2, participants were assessed for individual muscle stiffness (Stiff) via muscle natural oscillation. On session 3, participants were assessed for maximal StV on the forehand, backhand and serve actions. On session 4, participants were assessed using strength tests including bench press peak power (Wmax), maximal isometric strength (IsoMax) in 5 different positions, squat jump (SJ), countermovement jump (CMJ), and medicine ball overhead throw (MBT), following that order. Sessions 2, 3, and 4 were separate 2 h apart and session 1 was executed 24 h before. Players performed one tennis technical-tactical training session of 90 min between sessions 1 and 2 and ceased activity for at least 14 h before resuming the testing protocol. The use of pain-relieving strategies (e.g., foam rolling, massage, ice baths, etc.) was not allowed during testing in order to avoid interferences with the results. Players were allowed to consume water *ad libitum*. Isotonic and energetic drinks were not allowed during the tests. The order of the sessions was established this way with the intention of avoiding the influence of stiffness, strength and power testing on the StV protocol. In order to ensure a better precision and reproducibility of singles measurements, the intra-session reliability of stiffness, strength, power and StV values was determined using a test-retest design. Thus, the same testing procedure that was carried out in the current study was repeated twice in the strength and power (1RM, Wmax, IsoMax, SJ, CMJ, and MBT), three times in the StV (forehand, backhand, and serve) and five times in stiffness measurements in all tennis players. Two familiarization sessions of all the strength and power tests were completed during the 2 weeks prior to the application of the protocol.

### Procedures

#### 1-Repetition Maximum Estimation

Maximum dynamic strength was estimated in session 1 using the load-velocity relationship via a progressive load test (Jidovtseff et al., 2011). All subjects were tested in four progressive loads in the bench press exercise. The number of series performed were executed in the following manner; eight repetitions with 20 kg of load; eight repetitions with 30 kg; eight repetitions with 35 kg, and eight repetitions with 40 kg. Participants had 5 min rest between sets. For each load, players were indicated to raise the bar as quickly as possible without releasing it. If eight repetitions were not performed, as many as the subject performed were recorded. The test recorded only the concentric phase of the exercise so the bar had an initial position of 3 cm above the nipple line. During the whole movement, the subjects had their backs on the bench and their hips flexed at 90°. The best repetition was recorded for the analysis of the maximum speed achieved. 1RM was estimated using a regression line plotted through the known load (X) and average velocity (Y). From this linear regression, the slope, theoretical average velocity at 0 kg, and theoretical load at 0 m/s^–1^ were calculated by means of a linear transducer (CLTP, Chronojump Boscosystem^®^, Barcelona, Spain) (Jidovtseff et al., 2011). This encoder has been previously validated showing it has a valuable and reliable system for measuring movement velocity and for estimating power in strength and conditioning training exercises (Garnacho-Castaño et al., 2014).

#### Contractile Properties Measurement

**FIGURE 1:**
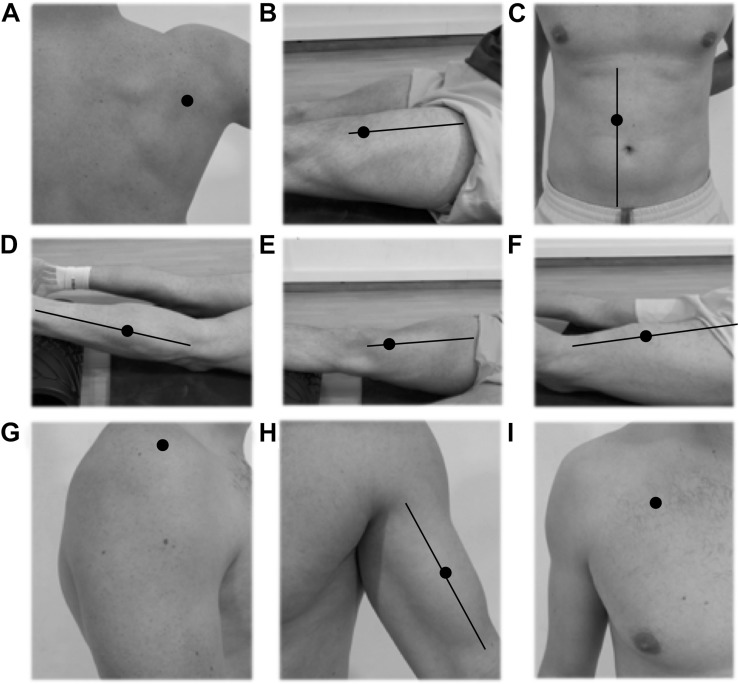
Mechanical muscle stiffness measurement spots. Myoton-Pro^®^ tip placement. **(A)** Infraspinatus. **(B)** Rectus femoris. **(C)** Rectus abdominis. **(D)** Gastrocnemius. **(E)** Vastus medialis. **(F)** Biceps femoris. **(G)** Deltoids. **(H)** Biceps brachii. **(I)** Pectoralis majoris.

In session 2, individual muscle stiffness was recorded on the dominant side of the body using a hand-held myometer (Myoton-Pro, Myoton AS, Tallinn, Estonia). The dominant extremity or side of the body was established based on upper body dominance. Before the assessment, body marks were established for the nine measurement points using the SENIAM electrode placement guides (Freriks et al., 1999; Konrad, 2005; Figure 1). The muscle groups chosen were those mostly involved in the hitting actions (Girard et al., 2005) attending to the whole kinetic chain; pectoralis major (PM); biceps brachii (B); infraspinatus (I); deltoids (D); rectus abdominis (RA), the rectus femoris (RF); vastus medialis (VM); biceps femoris (BF), and the lateral head of the gastrocnemius (G). The measurements were made with a state of muscle relaxation and the subjects lying down (RA), seated (PM, B, I, D) or in anatomical position (RF, VM, BF, G), depending on the test point. The tip of the Myoton-Pro was placed perpendicular to all measurement zones sampling at 15 ms with a force of 0.58 N and measured the damped natural oscillations cause by the probe impact. The device’s accelerometers operated at 3,200 Hz, offering an average value of five consecutive measurements. The Myoton-Pro reliability is expressed in Table 1, and shows excellent test-retest values (ICC = 0.95–0.99; CV = 0.3–0.9) as shown previously in other investigations (Zinder and Padua, 2013).

**TABLE 1:** Reliability of test measurements.

	ICC (95% CI)	CV (%)	SEM
SJ (cm)	0.889 (0.720–0.956)	3.1	2.3
CMJ (cm)	0.916 (0.787–0.967)	0.5	2.5
1 kg MBT (m)	0.870 (0.693–0.980)	3.1	0.9
2 kg MBT (m)	0.979 (0.956–0.991)	0.4	0.3
3 kg MBT (m)	0.976 (0.950–0.989)	1.3	0.3
WmaxBP (W)	0.915 (0.843–0.961)	3.5	46.1
IsoIR (N)	0.815 (0.544–0.925)	6.4	21.2
IsoAbd (N)	0.923 (0.811–0.969)	2.9	13.4
IsoAdd (N)	0.870 (0.681–0.947)	1.6	21.5
IsoWrF (N)	0.947 (0.869–0.978)	2.5	11.2
IsoWrE (N)	0.935 (0.847–0.973)	1.4	8.6
SV (km⋅h^–1^)	0.803 (0.183–0.986)	1.1	9.3
FV (km⋅h^–1^)	0.937 (0.829–0.987)	3.4	7.6
BV (km⋅h^–1^)	0.900 (0.672–0.988)	1.1	6.5
AbdStiff (N⋅m^–1^)	0.996 (0.993–0.998)	0.9	6.1
BStiff (N⋅m^–1^)	0.981 (0.965–0.991)	0.3	3.2
DStiff (N⋅m^–1^)	0.991 (0.983–0.996)	0.9	5.3
PMStiff (N⋅m^–1^)	0.992 (0.985–0.996)	0.4	3.6
IStiff (N⋅m^–1^)	0.956 (0.920–0.979)	0.3	6.5
BFStiff (N⋅m^–1^)	0.996 (0.992–0.998)	0.5	5.3
RFStiff (N⋅m^–1^)	0.994 (0.988–0.997)	0.8	4.5
VMStiff (N⋅m^–1^)	0.988 (0.978–0.994)	0.5	3.3
GStiff (N⋅m^–1^)	0.998 (0.996–0.999)	0.4	6.2

*ICC, intraclass correlation coefficient; CI, confidence interval; CV, coefficient of variation. SEM, standard error of measurement; StV, stroke velocity; SV, serve velocity; FV, forehand velocity; BV, backhand velocity; ITN, international tennis number; IsoIR, maximum isometric internal rotation strength; IsoAdd, maximum isometric adduction strength; IsoAbd, maximum isometric abduction strength; IsoWrF, maximum isometric wrist flexion strength; IsoWrE, maximum isometric wrist extension strength; WmaxBP, peak power bench press; MBT, medicine ball throw; SJ, squat jump; CMJ, countermovement jump; BStiff, biceps stiffness; PMStiff, pectoral majoris stiffness; DStiff, deltoids stiffness; IStiff, infraspinatus stiffness; AbdStiff, rectus abdominis stiffness; BFStiff, biceps femoris stiffness; RFStiff, rectus femoris stiffness; VMStiff, vastus medialis stiffness; GStiff, gastrocnemius stiffness.*

#### Maximum Hitting Velocity

In session 3, subjects performed a standardized warm-up that included mobility exercises, 5 min of free rallies and 5–10 progressive serves. Each subject executed a series of 6 flat services on each side of the court with 60 s of rest between sets and 12 forehands and 12 backhands (crossed-court) without alternating strokes. Only the serves that were in the serve box and the groundstrokes that landed in the court were counted. StV was determined using a hand-held radar gun [Stalker ATS II, United States, frequency: 34.7 GHz (Ka-Band) ± 50 MHz] and peak velocity was registered for further analysis. The radar was positioned in the center of the baseline, 2 m behind the line and at an approximate height of 2 m for the serves and behind the player following the trajectory of the ball for groundstrokes. Hitting as hard and precise as possible to the “T” was indicated when serving and cross-court when hitting groundstrokes. Immediate feedback was provided to the subjects to encourage maximum effort. To avoid variability performing groundstrokes, balls were fed by a ball-throwing machine (Pop-Lob Airmatic 104, France) at a constant speed (68.6 ± 1.9 km ^*h*–1^).

#### Strength and Power Measurements

In session 4, the participants were asked to perform five maximum isometric tests following a protocol similar to that offered by Baiget et al. (2016). The different positions tested were: internal shoulder rotation with the elbow and shoulder flexed 90° (IsoIR), horizontal shoulder abduction (IsoAbd), horizontal shoulder adduction (IsoAdd), wrist flexion (IsoWrF), and extension (IsoWrE). The test was performed sitting on an Ercolina machine (Technogym Company^®^, Cesena, Italy). The participants sat in a position with a 90° hip flexion and the back resting on a bench. All the participants were fastened with a harness on the chest to avoid unwanted movements. Only the dominant extremity was evaluated. The maximum isometric force peak was recorded using a strain gauge sampling at 80 Hz (Chronojump, Boscosystem^®^, Barcelona, Spain). Positions were established before each test using a goniometer. Subjects performed three maximal voluntary contractions for 3–6 s and spaced by 1-min rest between attempts. Regarding upper body, peak power (Wmax) was assessed with a linear transducer (CLTP, Chronojump, Boscosystem^®^, Barcelona, Spain) and analyzed with Chronojump Software (v1.8) using a load based on each participant’s 1RM. Each subject performed eight repetitions on the bench press exercise without verbal encouragement given. Following the literature (Soriano et al., 2017) the load was set at 50% of the 1RM since it seems closest to optimal for the development of the peak power in the bench press. Only the propulsive concentric phase of the exercise was analyzed. During the whole movement, the subjects had their backs on the bench and their hips flexed at 90°. No bouncing or arching the back was allowed. MBT were evaluated using 3 external loads of 1, 2, and 3 kg. The participants placed themselves behind a line and performed three throws with each of the balls, spaced by 1 min of rest between them. Throws had to be performed with both hands, above the head, without jumping or taking advantage of the momentum of the legs or falling with the feet in front of the throwing line. MBT seem to be useful for testing tennis players as they show high external validity, because they involve the coordination of body segments (i.e., kinetic chain) (Fernández-Fernández et al., 2014). Regarding lower body, CMJ and SJ in order to assess lower body power were performed on a contact platform (Chronojump, Boscosystem^®^, Barcelona, Spain). Each participant executed three maximum jumps spaced by 45 s of passive rest. The best trial (i.e., highest jump height) was used for the subsequent analysis.

### Statistical Analyses

The values presented are expressed as mean ± SD and 95% confidence intervals (95% CI). The normality of the distributions and homogeneity of variances were assessed with the Shapiro–Wilk test, all variables showed normal distributions except for ITN. The reliabilities of test measurements were assessed using intraclass correlation coefficients (ICCs), the standard error of measurements (SEM), and the coefficient of variation (CV). All of stiffness, strength, power and serve, forehand, and backhand velocity measurements reached an acceptable level of reliability and are presented in Table 1. Pearson correlation coefficients were used to examine the relations between serve, forehand and backhand velocity and strength, power, and stiffness variables. Strength, power and stiffness variables were correlated with the ITN of the players using Spearman rank order correlation. Correlations were classified as trivial (0–0.1), small (0.1–0.3), moderate (0.3–0.5), large (0.5–0.7), very large (0.7–0.9), nearly perfect (0.9), and perfect (1.0) (Hopkins et al., 2009). Statistical significance was accepted at an alpha level of *p* ≤ 0.05. All statistical analyses were performed using IBM SPSS Statistics 23.0 (SPSS, Inc., Chicago, IL, United States).

## Results

The correlation coefficients between strength, power and StV), and ITN are presented in Table 2. Correlations between muscle stiffness values, StV and ITN are presented in Table 3. Moderate inverse correlations were found between serve velocity (SV) and ITN (*r* = −0.43; *p* = 0.05), and large positive correlations between ITN and pectoralis majoris stiffness (PMStiff) (*r* = 0.53; *p* = 0.01) and isometric wrist flexion (IsoWrF) (*r* = 0.58; *p* = 0.006). Also, moderate inverse and positive correlations were observed between PMStiff and forehand velocity (FV) (*r* = −0.45; *p* = 0.03) and between gastrocnemius/infraspinatus stiffness (GStiff/IStiff) and SV (*r* = 0.45; *p* = 0.04, *r* = 0.42; *p* = 0.05), respectively. Regarding strength and power values, no significant correlations were found between upper or lower body values and hitting velocities.

**TABLE 2:** Strength and power variables and correlation coefficients (r) between maximal stroke velocity and competitive level (*n* = 21).

Variable	Mean performance	Maximal StV^§^			Competitive level^‡^
		SV (r)	FV (r)	BV (r)	ITN (r)
SV (Km⋅h^–1^)	179.5 ± 12	1	0.49^†^	0.31	−0.43^†^
FV (Km⋅h^–1^)	154.3 ± 11.6	0.49^†^	1	0.15	0.01
BV (Km h^–1^)	136.5 ± 7.8	0.31	0.15	1	–0.35
IsoIR (N)	176.8 ± 36.5	0.005	0.003	0.36	0.27
IsoAbd (N)	139.7 ± 33.4	0.15	–0.23	–0.10	0.19
IsoAdd (N)	209.8 ± 47.8	0.31	–0.06	0.16	–0.16
IsoWrF (N)	259.9 ± 68.2	–0.06	0.07	–0.18	0.58^†^
IsoWrE (N)	161.3 ± 53.1	–0.02	0.06	–0.19	0.28
WmaxBP (W)	503.6 ± 92.6	0.11	0.11	0.04	–0.08
1 kg MBT (m)	12.9 ± 1.5	0.24	0.001	0.16	–0.02
2 kg MBT (m)	9.4 ± 1.4	0.12	–0.05	0.04	0.01
3 kg MBT (m)	7.8 ± 1.2	0.21	0.002	0.01	0.03
SJ (cm)	27.4 ± 5.1	0.15	–0.05	0.30	–0.07
CMJ (cm)	30.1 ± 6.3	0.04	–0.003	0.28	0.35

*Values are mean ± SD. § Pearson product moment correlations. ^‡^Spearman correlation coefficients. StV, stroke velocity; SV, serve velocity; FV, forehand velocity; BV, backhand velocity; ITN, international tennis number; IsoIR, maximum isometric internal rotation strength; IsoAdd, maximum isometric adduction strength; IsoAbd, maximum isometric abduction strength; IsoWrF, maximum isometric wrist flexion strength; IsoWrE, maximum isometric wrist extension strength; WmaxBP, peak power bench press; MBT, medicine ball throw; SJ, squat jump; CMJ, countermovement jump. ^†^p < 0.05.*

**TABLE 3:** Individual muscle stiffness variables and correlation coefficients (r) between maximal stroke velocity and competitive level (*n* = 21).

Variable	Mean performance	Maximal StV^§^			Competitive level^‡^
		SV (r)	FV (r)	BV (r)	ITN (r)
BStiff	207.1 ± 21.5	–0.14	–0.21	–0.13	0.31
PMStiff	235.6 ± 39.4	–0.29	−0.45^†^	–0.16	0.53^†^
DStiff	223.0 ± 53.4	–0.01	–0.38	–0.16	–0.16
IStiff	246.2 ± 51.1	0.42	0.15	0.16	0.19
AbdStiff	329.6 ± 87.8	–0.11	0.11	–0.10	–0.17
BFStiff	394.9 ± 79.6	0.17	–0.12	0.24	–0.04
RFStiff	318.2 ± 50.5	0.14	–0.07	0.10	–0.07
VMStiff	218.9 ± 30.7	–0.10	–0.22	–0.01	0.03
GStiff	31.5 ± 198.9	0.45^†^	0.02	–0.06	–0.08

*Values are mean ± SD. § Pearson product moment correlations. ^‡^Spearman correlation coefficients. StV, stroke velocity; SV, serve velocity; FV, forehand velocity; BV, backhand velocity; ITN, international tennis number; BStiff, biceps stiffness; PMStiff, pectoral majoris stiffness; DStiff, deltoids stiffness; IStiff, infraspinatus stiffness; AbdStiff, rectus abdominis stiffness; BFStiff, biceps femoris stiffness; RFStiff, rectus femoris stiffness; VMStiff, vastus medialis stiffness; GStiff, gastrocnemius stiffness. ^†^p < 0.05.*

## Discussion

This study aimed to analyze strength (maximum isometric strength) and power (medicine ball throws, SJ, CMJ, and bench press peak power) characteristics alongside ITN as possible determinants of StV, including serve and groundstrokes, in junior tennis players. The main finding was that an increased gastrocnemius (GStiff), infraspinatus (IStiff), and decreased pectoralis majoris stiffness (PMStiff) may have some positive influence over performance in serve (SV) and forehand velocity (FV) respectively. Also, SV was inversely correlated to ITN. Moreover, strength and power values proved to be weak predictors of StV in this particular sample of junior tennis players. These results indicate that players of these characteristics that are able to reach higher velocity production in the serve and groundstrokes don’t specifically rely on the assessed strength and power characteristics.

In other investigations it has been shown that physical aspects such as strength and power are determinant for producing ball velocity (Fett et al., 2018; Palmer et al., 2018), also when comparing players of different levels (Girard et al., 2005; Ulbricht et al., 2016). Non-significant positive results have been found between some physical indicators such as isokinetic strength and SV (Ellenbecker, 1991) but recent findings restate the importance of strength and power characteristics for velocity production on both, national young tennis players (Fett et al., 2018) and highly competitive players (Baiget et al., 2016). These differences in results with the present study could be explained by the variance of the analyzed subjects. The cited investigations carried out assessments with highly skilled players that respond to elite population (Baiget et al., 2016; Fett et al., 2018). Added to the fact that the players participating in this study where of a different level (ITN 2/4 vs. 1/2) than those present in other investigations (Baiget et al., 2016), there could also be an influence due to the age (17.0 ± 0.8 vs. 9.4–17.9 age range) (Fett et al., 2018) of the subjects for contrary results. Younger players may still rely more thoroughly on technique and coordinative skills while serving or hitting rather than on strength values that may become more important as both age and level increases. As suggested in literature, this may indicate that although SV is highly related to tennis performance, velocity production may depend more importantly on strength and power parameters as the player grows and the performance level raises (Girard, 2009).

Studies focused on SV have generally established positive results between MBT and velocity production in young tennis players (Fett et al., 2018). MBT have even been established as fundamental indicators of whole-body explosive power regardless of throwing technique (Fernández-Fernández et al., 2014). Surprisingly, no correlations were found between the overhead MBT and StV in this study. Leaving aside the lack of positive results between MBT and SV, results regarding FV and BV may be explained by methodological issues. MBT testing focused on the overhead motion only, and, although the conducted test assessed three different loads (1, 2, and 3 kg), it did not contemplate mimicking the forehand or the backhand motion (i.e., throwing the medicine ball with one or two hands from the side of the body), as previously studied and found positively correlated with SV (Ulbricht et al., 2016; Fett et al., 2018). On the other hand, consistent with findings in other studies (Kraemer et al., 2003), poor correlations were found for the bench press exercise and either groundstrokes or SV. This, most likely, is explained by the lack of movement similarity and, unlike the MBT testing, the low specificity of the action. Also, muscle involvement in the bench press exercise is reduced to fewer groups than in tennis specific strokes.

Regarding maximal isometric values, no correlations with any of the actions measured were found besides results indicating a positive association between maximal isometric wrist flexion and a higher ITN score. Previous studies have positively correlated isometric strength values with throwing (Ferragut et al., 2011; Freeston et al., 2016) or even tennis specific motions in both, upper and lower body (Fett et al., 2018; Hayes et al., 2018). The findings in this investigation are consistent with those present in other works that found no relation between isometric measurements and tennis actions (Bonato et al., 2015). However, and given that the positions measured are rather different than those focused on the grip, results are surprising. Due to the similarity of the positions tested and those present and involved in the kinetic chain at the wrist, elbow and shoulder, it was expected to obtain certain positive relations between both variables. As literature points out, increased levels of maximal isometric strength, evaluated during multi-joint actions, are likely to be positively related to dynamic performances (Juneja et al., 2010) such as the serve and groundstrokes (Baiget et al., 2016). However, the discrepancy could be explained by the fact that the participants were similar in age but not in level (ITN 1–2 vs. ITN 2–4 or high-performance vs. elite) to those present in other studies (Baiget et al., 2016; Hayes et al., 2018). It would be possible that these players still rely more on coordinative aspects while serving or hitting rather than on strength values that may increase with age and level (Girard, 2009). Moreover, the ability to apply a high amount of force in a short time (i.e., rate of force development) could be of greater importance over absolute values of strength when referring to explosive dynamic actions such as the analyzed strokes.

Lower body power variables analyzed (i.e., SJ and CMJ) did not correlate with any of the StV variables. Although the contribution of the legs is considered widely as one of the main parameters supporting the effectiveness of the kinetic chain (Kibler, 2009), these results indicate that strength values such as explosiveness and power may not be as determinant as coordinative aspects involved in tennis strokes (Bonato et al., 2015; Dossena et al., 2018). Strong consistency has been found in other investigations stating vertical jumps as predictors of sprinting times and lower body strength/power values in tennis players (Kraemer et al., 2003; Girard et al., 2005). Nevertheless, regarding actions such as strokes, this does not seem as clear. As predominantly lower body actions could benefit from enhanced strength and power values, regarding upper body actions such as serves and groundstrokes, legs might provide a coordinative and timing contribution to velocity-production (Bonato et al., 2015; Dossena et al., 2018; Fett et al., 2018).

Regarding muscle stiffness, results indicate moderate positive correlations between GStiff, IStiff and SV, PMStiff, and ITN, and inverse correlations between PMStiff and FV. As far as we know, no previous studies have attempted to correlate individual stiffness values and StV, although in other investigations, some findings indicate the importance of stiffness when we refer to actions that rely on the SSC (Brughelli and Cronin, 2008; Pruyn et al., 2014; Kalkhoven and Watsford, 2018; Sheehan et al., 2018). Generally, literature has found positive evidence linking greater stiffness values to enhanced sprinting or jumping, mainly lower body actions (Brughelli and Cronin, 2008; Kalkhoven and Watsford, 2018). This happens because of the athlete’s capacity to store more elastic energy during ground contact and generate greater force output at push-off, increasing jump height and running speed (Brazier et al., 2017). On the other hand, in predominantly upper body motions as tennis strokes, research is scarce. The study carried out by Sheehan et al. (2018), found a strong relationship between vertical stiffness assessed via a unilateral leg hop test and the club head speed in male golfers. When analysing upper body muscle groups, no significant results regarding pectoralis majoris, latissimus dorsi, flexor carpi ulnaris, and club head speed were found. No measurements of vertical stiffness were included in the study design of this investigation, limiting the findings regarding the influence of muscle stiffness of the lower body and its relation to velocity production. Nevertheless, the strong and consistent correlations between stiffness and dynamic performance observed in other investigations (Sheehan et al., 2018), may indicate that greater stiffness values in lower body muscles could be beneficial for performance in motions taking place predominantly in the upper body. In this line, the fact that a higher level of stiffness of the gastrocnemius benefits SV could follow the same idea, as energy storage and transfer from lower to upper body is key for tennis actions (Kibler, 2009). Regarding purely upper body muscle stiffness values, results are similar to those seen in other studies (Sheehan et al., 2018), observing small correlations with StV. Greater levels of PMStiff seem to have a certain negative influence on FV and ITN, suggesting that a greater level of stiffness in the upper body, far from being beneficial could interpose velocity production in junior tennis players. This matches findings in literature (Sheehan et al., 2018), suggesting that tendencies for compliancy might be favorable for motions involving the SSC in the upper body. As complex motor skills such as the tennis groundstrokes rely, among other aspects, on the principle of coordination of individual impulses and an effective kinetic chain (Kibler, 2009, 2014), high levels of upper body stiffness could be counterproductive for these particular actions, affecting execution. Moreover, tightness and increased external rotation when compared to the non-dominant side have been well established as contributors of shoulder internal rotation deficit (Marcondes et al., 2013), which generally can lead to shoulder injury in overhead athletes (Moreno-Pérez et al., 2015). It could be, as it appears when speaking of lower body actions, that an increased or non-sufficient level of stiffness could contribute to a greater injury risk, due to increased shock, peak forces and reduced ROM (Brazier et al., 2017). As a general idea, stiffness values may be beneficial to reduce electromechanical delay and enhance rate of force development, as could be the case of moderate positive correlations found here between IStiff and SV. On the other hand, stiffness could interfere in technical parameters that are key for velocity production in complex tennis strokes and increase injury likelihood due to restrictions in ROM. In any case, this is speculative and additional work is required to state a conclusion on the matter. The fact that stiffness measures were collected individually and in a relaxed state that differs highly with that present during competition may be a reason for generally poor correlations found in this study. Therefore, future investigations may explore upper body stiffness in a more “global” manner, as it has generally been done concerning lower body (i.e., hopping tests) and try to measure muscle stiffness in different contraction regimes.

This study showed some limitations. Maximal speed measurements, especially in groundstrokes, don’t take into account technical and tactical aspects on which skilled players may rely on in order to reach greater performance (i.e., spin or shot placement).

## Practical Applications

As specific values of stiffness remain unclear, this study suggests practitioners include control and evaluation of stiffness as it may have influence in performance or injury risk. Moreover, due to multiple aspects affecting StV, designing programs that include technical and tactical assessment alongside strength and power enhancement, coordinative training and biomechanical aspects seems essential to enhance velocity production. Performance in these actions are affected by several aspects and the influence of them over StV may vary depending on the athlete’s age and level.

## Conclusion

In conclusion, an increased GStiff and IStiff seem to correlate to greater SV and high values of PMStiff affect negatively the player’s FV. Greater stiffness values of the gastrocnemius may enhance StV, possibly supporting power transfer from lower to upper body. On the other hand, enhanced levels in muscles surrounding the shoulder complex could interfere in technical parameters that are key for velocity production in complex tennis strokes. Also, SV is inversely correlated to ITN, indicating that players with a higher number in this rating seem to serve faster. Moreover, strength and power values proved to correlate poorly to StV in this particular sample of junior tennis players. Results indicate that athletes of these characteristics that are able to reach higher velocity production in the serve and groundstrokes don’t specifically rely on the assessed strength and power characteristics, possibly due to the multifactorial nature of tennis strokes and the possibility that they become more important as age and level increase.

## Data Availability Statement

All datasets generated for this study are included in the article/supplementary material.

## Ethics Statement

The studies involving human participants were reviewed and approved by Catalan Sports Council Research Committee. Written informed consent to participate in this study was provided by the participants’ legal guardian/next of kin.

## Author Contributions

JC, EB, and FC contributed to the conceptualization, methodology, and review. EB contributed to the statistical analysis. JC contributed to the writing of the original draft preparation.

## Conflict of Interest

The authors declare that the research was conducted in the absence of any commercial or financial relationships that could be construed as a potential conflict of interest.
